# Higher testosterone is associated with increased inflammatory markers in women with SARS-CoV-2 pneumonia: preliminary results from an observational study

**DOI:** 10.1007/s40618-021-01682-6

**Published:** 2021-11-03

**Authors:** V. Di Stasi, G. Rastrelli, F. Inglese, M. Beccaria, M. Garuti, D. Di Costanzo, F. Spreafico, G. Cervi, G. F. Greco, A. Pecoriello, T. Todisco, S. Cipriani, E. Maseroli, I. Scavello, C. Glingani, M. Franchini, M. Maggi, G. De Donno, L. Vignozzi

**Affiliations:** 1 Andrology, Women’s Endocrinology and Gender Incongruence Unit, Department of Experimental and Clinical Biomedical Sciences “Mario Serio”, University of Florence, Careggi Hospital, Viale Pieraccini, 6, 50134 Florence, Italy; 2 Intensive Care Respiratory Unit, Carlo Poma Hospital, Mantova, Italy; 3 Department of Hematology and Transfusion Medicine, Carlo Poma Hospital, Mantova, Italy; 4 Endocrinology Unit, Department of Experimental and Clinical Biomedical Sciences “Mario Serio”, University of Florence, Careggi Hospital, Viale Pieraccini, 6, Florence, Italy

**Keywords:** COVID-19, Inflammatory markers, Gender difference, Prognosis, Sex hormones

## Abstract

**Purpose:**

Objective of this study was to assess the association between testosterone (T) levels and biochemical markers in a cohort of female patients admitted for SARS-CoV-2 infection in a respiratory intensive care unit (RICU).

**Methods:**

A consecutive series of 17 women affected by SARSCoV-2 pneumonia and recovered in the RICU of the Hospital of Mantua were analyzed. Biochemical inflammatory markers as well as total testosterone (TT), calculated free T (cFT), sex hormone-binding globulin (SHBG), and luteinizing hormone (LH) were determined.

**Results:**

TT and cFT were significantly and positively associated with PCT, CRP, and fibrinogen as well as with a worse hospital course. We did not observe any significant association between TT and cFT with LH; conversely, both TT and cFT showed a positive correlation with cortisol. By LOWESS analysis, a linear relationship could be assumed for CRP and fibrinogen, while a threshold effect was apparent in the relationship between TT and procalcitonin, LDH and ferritin. When the TT threshold value of 1 nmol/L was used, significant associations between TT and PCT, LDH or ferritin were observed for values above this value. For LDH and ferritin, this was confirmed also in an age-adjusted model. Similar results were found for the association of cFT with the inflammatory markers with a threshold effect towards LDH and ferritin with increased LDH and ferritin levels for values above cFT 5 pmol/L. Cortisol is associated with serum inflammatory markers with similar trends observed for TT; conversely, the relationship between LH and inflammatory markers had different trends.

**Conclusion:**

Opposite to men, in women with SARS-CoV-2 pneumonia, higher TT and cFT are associated with a stronger inflammatory status, probably related to adrenal cortex hyperactivity,

## Introduction

The new severe acute respiratory syndrome coronavirus 2 (SARS-CoV-2) infection has spread rapidly around the world in the last few months, thus resulting in more than 106 million persons globally infected and 2316 million deaths as of February 9, 2021 (https://covid19.who.int/).

The clinical manifestations of SARS-CoV-2 infection are extremely different, encompassing asymptomatic infections but also life-threatening Acute Respiratory Distress Syndrome (ARDS). This clinical heterogeneity and the rapid evolution of this new pandemic syndrome made urgent the identification of pathogenic mechanisms underlying the heterogeneity and possible risk factors [1–3].

Clinical and epidemiological studies have identified age and male gender as substantial risk factors for COVID-19 poor prognostic clinical outcomes and higher mortality rate [4–6]. Accordingly, an independent and internationally recognized initiative for gender equality (Global Health 50/50), reported COVID-19 sex disaggregated data by showing a higher case fatality rate in men vs. women in all the age-bands [7]. Therefore, among the potential gender-specific mechanistic differences that make males more susceptible to severe COVID-19, a likely role for sex hormones have been recognized. In particular, testosterone has been proposed as a modulator of the inflammation underlying the SARS CoV-2 infection [4, 8–10]. Interestingly, at the edge of the first wave of the epidemic in Italy, we reported that, among a sample of 31 men recovered in Respiratory Intensive Care Unit (RICU) for ARDS due to SARS-CoV-2, low testosterone (T) levels mirrored a more severe inflammatory status and were able to predict adverse clinical outcomes [10]. Specifically, we found that total T (TT) below 5 nmol/L and calculated free T (cFT) below 100 pmol/L were associated with a steep increase of circulating neutrophil, procalcitonin (PCT) and lactate dehydrogenase (LDH), while only TT was also negatively associated with C-reactive protein, CRP, and ferritin levels. In addition, TT < 5 nmol/L and cFT < 100 pmol/L were associated with a 20–40% lower likelihood of improving their clinical conditions. This evidence was perfectly in line with the well-documented protective effects of testosterone in several inflammatory diseases. Epidemiologically, an age-related androgen decline in men has been correlated with the clinical course, in terms of either outcomes or comorbidities, of several inflammatory and autoimmune diseases [11].

Although supported by a weaker evidence, an immunomodulatory effect of T is also documented in women [12, 13]. To assess the role of this hormone in SARS-CoV-2 women and compare it to findings in the male counterpart, we collected data on T levels in female patients from the same clinical setting.

Thereof, we sought to perform a preliminary study to assess the association between T levels and biochemical markers in a cohort of female patients admitted for SARS-CoV-2 infection in the RICU of a single Hospital in Mantua, one of the epicenters of the SARS-CoV-2 epidemic in Italy. This study reports the findings obtained on a small sample of 17 patients with SARS-CoV-2 infection followed to the discharge to lower intensity care units or death.

## Methods

Data from a consecutive series of 17 female patients with SARS-CoV-2 pneumonia and recovered in the respiratory intensive care unit (RICU) of the “Carlo Poma” Hospital in Mantua, Italy, were analyzed. A laboratory pharyngeal-nose swab positivity of SARS-CoV-2 infection was confirmed by chest X-ray. Acute respiratory distress syndrome (ARDS) was defined by the “Berlin definition,” and patients were segregated into mild ARDS (PaO_2_/FiO_2_ ≤ 300 mmHg and > 200 mmHg), moderate ARDS (PaO_2_/FIO_2_ ≤ 200 mmHg and > 100 mmHg), and severe ARDS (PaO_2_/FiO_2_ ≤ 100 mmHg) (Definition Task Force ARDS, 2012).

According to the protocol (approved by the Local Ethics Committee Val Padana, Mantua, Italy) and as for clinical practice, each patient underwent a standardized diagnostic workup. Specifically, blood samples were drawn on the first morning after the admission to the RICU within 8.00 AM, in fasting condition, for the determination of blood count and leukocyte formula, creatinine, uric acid, electrolytes, transaminases, albumin, creatine phosphokinase (CPK), C-reactive protein (CRP), procalcitonin (PCT), lactate dehydrogenase (LDH), ferritin, D-dimer, fibrinogen, interleukin 6 (IL-6), TT, sex hormone–binding globulin (SHBG), cortisol and luteinizing hormone (LH). Hematological and biochemical analyses were performed in the central laboratory of the “Carlo Poma” Hospital (Mantua, Italy) with commercially available kits routinely used for hospital clinical practice. TT was measured once by an immunoassay (Electrochemiluminescence Immunoassay, ECLIA), and free T was calculated by the Vermeulen formula [14]. Cortisol was measured only in patients (*n* = 12) not receiving steroid therapy.

### Statistical methods

Data were expressed as medians [interquartile ranges] or percentage for continuous and categorical variables, respectively. Univariate relationships among T and biochemical markers were firstly assessed by Spearman's rank correlation. Afterwards, unadjusted and age-adjusted linear regression models were carried out. Non-linearity of the relationships were explored by the locally weighted scatterplot smoothing (LOWESS) analysis and formally checked by linear regression models with linear spline functions for T. This analysis allowed identifying threshold levels at which a significant change in the slope of the association between T and other blood markers occurred.

All the analyses were performed by Statistical Package for the Social Sciences Statistics 26 (IBM Corporation). Spline linear functions were carried out by Stata MP 13 (StataCorp).

## Results

Table 1 details descriptive statistics of the cohort including information collected at the admission to RICU. Of the 17 patients only one was not in menopause. She was 50 years old and her hormone values on the first morning after RICU admission were as follow: TT = 0.52 nmol/l, albumin = 3.12 g/dL, SHBG = 97.8 nmol/l, cFT = 4.3 pmol/l, LH = 8.9 mU/l, cortisol = 18.5 mcg/dL. She was discharged from RICU after 26 days of hospitalization for better clinical conditions. The relationship between T and biochemical inflammatory markers was firstly assessed by univariate correlation tests. Table 2 shows that TT and cFT measured on the first morning after RICU admission were significantly and positively associated with inflammatory markers assessed at the same time point, including procalcitonin, CRP, and fibrinogen. Accordingly, higher TT and cFT were related with a worse hospital course, being associated with longer hospitalization in RICU (where the present observations were collected) and ICU (transferred from RICU for deteriorated clinical conditions).

**Table 1:** Descriptive statistics of the cohort

	Reference range	Values in the cohort (*n* = 17)
Demographics and previous medical history		
Age (years)	–	69.0 [57.5–74.0]
Menopause (%)	–	94.1%
Smoking habits (%)		
Former smoker	–	11.8
Current smoker	–	11.8
Obesity (%)	–	52.9
Hypertension (%)	–	76.5
ACEi (%)		17.6
ARBs (%)		35.3
Dyslipidemia (%)	–	17.6
Diabetes (%)	–	29.4
Hypothyroidism (%)	–	5.9
Chronic Renal Failure (%)	–	0.0
Arrhythmia (%)	–	5.9
Psychiatric Diseases (%)	–	11.8
Hematologic Diseases (%)	–	11.8
CVD (%)	–	11.8
Liver Diseases (%)	–	0.0
Parameters during Hospitalization in RICU		
Time in RICU(days)	–	6.0 [3.5–12.5]
PaO_2_/FiO_2_ (mm Hg)	< 300 for ARDS	152.0 [113.5–178.1]
Severe ARDS (%)	PaO2/FiO2 ≤ 100 mm Hg	17.6
Moderate ARDS (%)	PaO2/FiO2 100–200 mm Hg	82.4
WBC (10^3^/µL)	4.4–11	8.4 [5.3–11.1]
Neutrophils (10^3^/µL)	2.0–7.5	6.6 [3.6–9.0]
Lymphocytes (10^3^/µL)	1.3–4.8	1.3 [0.8–1.7]
Hemoglobin (g/dl)	13.5–17–5	11.2 [10.0–11.9]
Hematocrit (%)	40–50	35.8 [33.1–37.4]
Platelets (10^3^/µL)	150–400	356.0 [253.5–449.0]
Creatinine (mg/dl)	0.6–1.3	0.7 [0.6–1.0]
Uric acid (mg/dl)	2.5–7.2	4.3 [3.0–6.9]
Sodium (mEq/L)	135–145	139.0 [137.0–141.0]
Potassium (mEq/L)	3.4–4.7	3.6 [3.4–3.7]
AST (U/L)	10–33	28.0 [19.5–31.8]
ALT (U/L)	5–37	21.0 [11.5–33.5]
CPK (U/L)	25–200	50.0 [38.5–75.5]
CRP (mg/L)	0–5	25.6 [15.8–62.5]
Procalcitonin (ng/ml)	0–0.09	0.05 [0.05–0.22]
LDH (U/L)	150–450	471.0 [420.0–528.0]
Fibrinogen (mg/dl)	150–450	467.5 [331.5–643.5]
D-Dimer (ng/ml)	< 500	1712.0 [987.5–3683.0]
Ferritin (ng/ml)	30–400	400.0 [233.0–967.5]
IL-6 (pg/ml)	< 7	71.7 [55.0–109.5]
TT (nmol/L)	0–1.5	0.5 [0.2–1.2]
cFT(pmol/L)	–	5.2 [2.8–15.1]
SHBG (nmol/L)	27.1–128.0	32.7 [27.4–54.5]
LH (U/L)	7.7–58.5	16.7 [4.3–27.0]
Cortisol (μg/dl)	6.0–18.4	20.1 [14.3–24.8]

Data are reported as median and interquartile range for continuous variables and as percentage for categorical variables

*ARDS* acute respiratory distress syndrome, *AST* alanine transaminase, *AST* aspartate transaminase, *CPK* creatine phosphokinase, *CRP* C-reactive protein, *CVD* cardiovascular disease, *IL-6* interleukin 6, *LDH* lactate dehydrogenase, *LH* luteinizing hormone, *RICU* respiratory intensive care unit, *SHBG* sex hormone–binding globulin, *T* testosterone, *FAI* Free Androgen Index, *WBC* white blood cell

**Table 2:** Correlation between total and free testosterone with hormones, biochemical inflammatory markers and days in RICU

	Total testosterone (nmol/l)		Calculated free testosterone (pmol/l)	
	Spearman’s *ρ*	*p*	Spearman’s *ρ*	*p*
Procalcitonin (ng/ml)	0.727	**0.001**	0.710	**0.003**
LDH (U/L)	0.396	0.115	0.333	0.208
Ferritin (ng/ml)	0.454	*0.067*	0.294	0.268
CRP (mg/l)	0.673	**0.003**	0.647	**0.007**
Fibrinogen (mg/dl)	0.669	**0.005**	0.513	**0.042**
Days in RICU	0.571	**0.017**	0.379	0.148
Days in ICU	0.593	**0.012**	0.493	*0.052*
LH (U/L)	− 0.317	0.232	-0.280	0.294
Cortisol (µg/dl)	0.954	** < 0.0001**	0.842	**0.002**

Bold: Statistically significant

Italic: not fully significant

Data derived from Spearman’s rank correlation test

*LDH* lactate dehydrogenase, *CRP* C-reactive protein, *RICU* respiratory intensive care unit, *ICU* intensive care unit, *LH* Luteinizing Hormone

To further explore the relationship of T with adverse biochemical and clinical parameters in women with SARS-CoV-2 pneumonia, we checked the relationship of TT or cFT with the releasing hormone (LH) and, surprisingly, we did not observe any significant association (Table 2). On the contrary, both TT and cFT showed a strong positive correlation with cortisol level (Table 2), which, in turn, was negatively associated with LH (*ρ* = − 0.748, *p* = 0.013). None of the aforementioned hormones correlated significantly with age (*ρ* = − 0.205, 0.132, − 0.093, − 0.024; *p* = 0.429, 0.626, 0.732, 0.947, for TT, cFT, LH and cortisol, respectively).

**Fig. 1:**
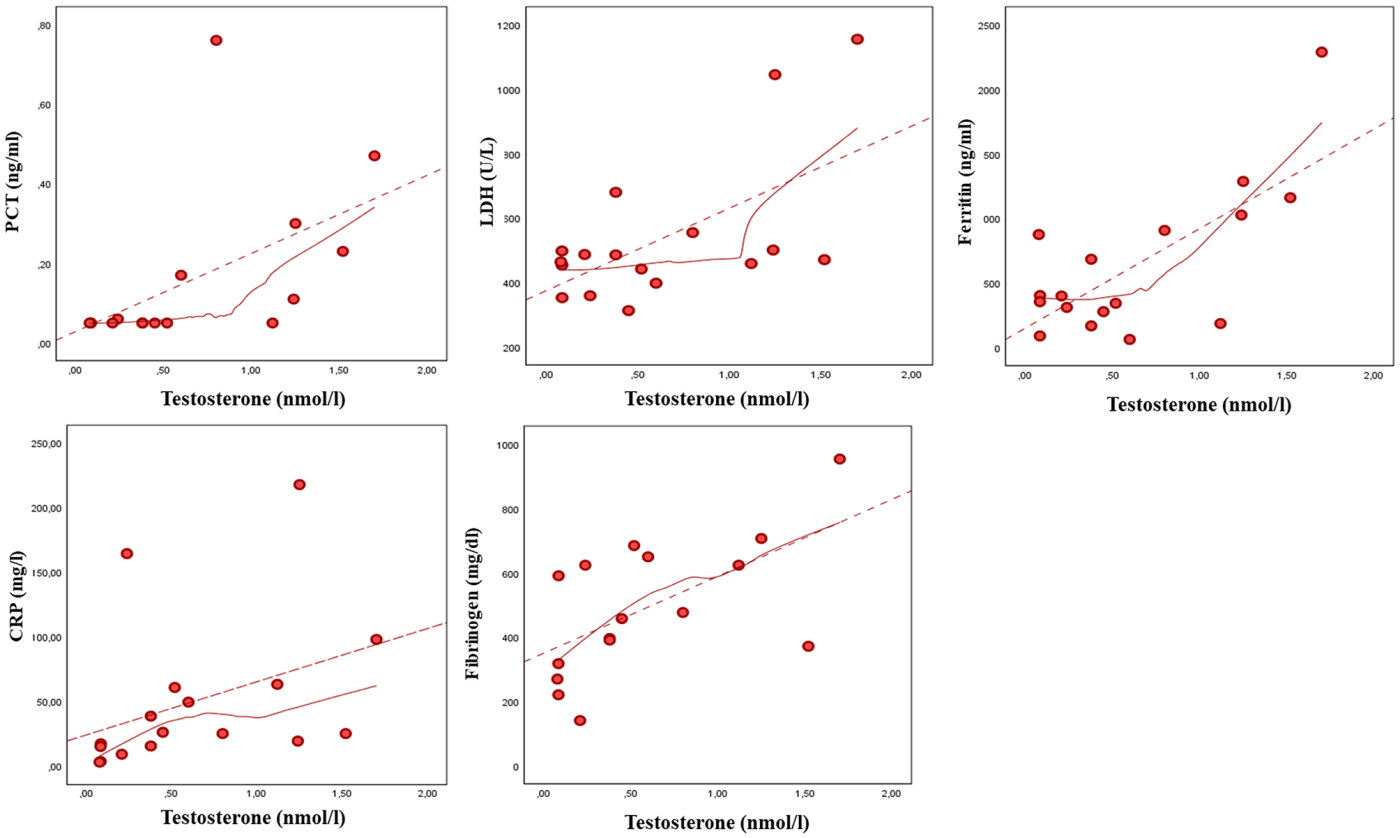
Relationship between total testosterone and biochemical inflammatory markers of severity of SARS-CoV-2 pneumonia. The smooth curves were carried out by locally weighted scatterplot smoothing (LOWESS) analysis. *PCT* procalcitonin, *LDH* lactate dehydrogenase, *CRP* C-reactive protein

To assess the possible non-linearity of the relationship between androgens and the inflammatory markers, we conducted LOWESS analyses. Figure 1 suggests that, while a linear relationship could be assumed for CRP and fibrinogen, a threshold effect is apparent in the relationship between TT and procalcitonin, LDH and ferritin. Data from spline regression analyses are shown in Table 3. When the TT threshold value of 1 nmol/L was used, significant associations between TT and PCT, LDH or ferritin were observed for values above but not below this value. This was confirmed for the relationship between TT and LDH or ferritin in an age-adjusted model (Table 3). Similar results were found for the association of cFT with the inflammatory markers with a threshold effect towards LDH and ferritin with significantly increased LDH and ferritin levels for values above—but not below—cFT 5 pmol/L (Table 3). The number of days spent in ICU but not in RICU showed a linear association with TT (*B* = 7.99 [1.95;14.02], *p* = 0.013 and 4.66 [− 3.10;12.43], *p* = 0.219, for ICU and RICU, respectively) and cFT (*B* = 0.22 [0.02;0.41], *p* = 0.035 and 0.09 [− 0.28;0.48], *p* = 0.584, for ICU and RICU, respectively), even after adjusting for age.

**Table 3:** Association between total and free testosterone with biochemical inflammatory markers

	Unadjusted model			Age-adjusted model		
	Total testosterone					
	Linear	Non linear		Linear	Non linear	
		< 1 nmol/L	≥ 1 nmol/L		< 1 nmol/L	≥ 1 nmol/L
Procalcitonin (ng/mL)	***B* = 0.2**	*B* = 0.5	***B* = 0.6**	***B* = 0.2**	*B* = 0.3	*B* = 0.1
	**[0.0;0.4]**	[– 0.1;1.3]	**[0.1;1.1]**	**[0.0;0.4]**	[– 0.2;0.7]	[– 0.7;0.8]
	***p* = 0.036**	*p* = 0.076	**0.016**	***p* = 0.044**	*p* = 0.196	*p* = 0.827
	*R*^2^ = 0.277	*R*^2^ = 0.287		*R*^2^ = 0.279	*R*^2^ = 0.287	
LDH (U/L)	***B* = 255.7**	*B* = 42.0	***B* = 675.9**	***B* = 264.3**	*B* = 21.1	***B* = 768.2**
	**[69.7;441.7]**	[– 325.2; 409.3]	**[21.9;1329.8]**	**[67.3;461.4]**	[– 355.2;397.3]	**[67.4;1468.9]**
	***p* = 0.010**	*p* = 0.810	***p* = 0.044**	***p* = 0.012**	*p* = 0.905	***p* = 0.034**
	*R*^2^ = 0.364	*R*^2^ = 0.445		*R*^2^ = 0.373		
Ferritin (ng/mL)	***B* = 771.8**	*B* = – 62.6	***B* = 2412.6**	***B* = 727.1**	*B* = – 41.4	***B* = 2318.9**
	**[368.8;1174.9]**	[– 713.9;588.6]	**[1253.0;3572.2]**	**[314.2;1139.9]**	[– 722.0;639.2]	**[1051.2;3586.6]**
	***p* = 0.001**	*p* = 0.840	***p* = 0.001**	***p* = 0.002**	*p* = 0.898	***p* = 0.002**
	*R*^2^ = 0.526	*R*^2^ = 0.723		*R*^2^ = 0.563	*R*^2^ = 0.729	
CRP (mg/L)	*B* = 41.2	*B* = 57.2	*B* = 9.9	*B* = 46.7	*B* = 50.7	*B* = 38.3
	[– 14.8;97.3]	[– 60.9;175.3]	[– 200.4;220.2]	[– 11.3;104.7]	[– 70.6;172.0]	[– 187.6;264.2]
	*p* = 0.138	*p* = 0.317	*p* = 0.921	*p* = 0.106	*p* = 0.383	*p* = 0.720
	*R*^2^ = 0.141	*R*^2^ = 0.147		*R*^2^ = 0.192	*R*^2^ = 0.192	
Fibrinogen (mg/dL)	***B* = 239.3**	*B* = 292.3	*B* = 140.1	***B* = 218.5**	*B* = 339.4	*B* = – 19.2
	**[56.3;422.3]**	[– 96.3;680.8]	[– 522.0;802.3]	**[36.7;400.2]**	[– 39.6;718.5]	[– 694.2;655.8]
	***p* = 0.014**	*p* = 0.128	*p* = 0.655	***p* = 0.022**	*p* = 0.075	*p* = 0.952
	*R*^2^ = 0.360	*R*^2^ = 0.365		*R*^2^ = 0.441	*R*^2^ = 0.469	

	Calculated free testosterone					
	Linear	Non linear		Linear	Non linear	
		< 5 pmol/L	≥ 5 pmol/L		< 5 pmol/L	≥ 5 pmol/L
Procalcitonin (ng/mL)	*B* = 0.0	*B* = 0.0	*B* = 0.0	*B* = 0.0	*B* = 0.0	*B* = 0.0
	[– 0.0;0.0]	[– 0.1;0.1]	[– 0.0;0.0]	[– 0.0;0.0]	[− 0.1;0.1]	[− 0.0;0.0]
	*p* = *0.057*	*p* = 0.568	*p* = 0.133	*p* = 0.062	*p* = 0.549	*p* = 0.145
	*R*^2^ = 0.252	*R*^2^ = 0.262		*R*^2^ = 0.263	*R*^2^ = 0.276	
LDH (U/L)	***B* = 13.9**	*B* = – 6.2	***B* = 15.0**	***B* = 13.9**	*B* = -6.2	***B* = 15.0**
	**[6.1;21.6]**	[– 90.0; 77.6]	**[5.7;24.2]**	**[5.9;22.0]**	[− 93.5;81.2]	**[5.4;24.7]**
	***p* = 0.002**	*p* = 0.875	***p* = 0.004**	***p* = 0.003**	*p* = 0.881	***p* = 0.005**
	*R*^2^ = 0.512	*R*^2^ = 0.522		*R*^2^ = 0.519	*R*^2^ = 0.528	
Ferritin (ng/mL)	***B* = 34.2**	*B* = – 44.4	***B* = 38.5**	***B* = 35.0**	*B* = − 43.4	***B* = 39.3**
	**[15.0;53.5]**	[–24.91;160.3]	**[16.0;61.0]**	**[17.7;52.3]**	[− 226.2;139.5]	**[19.1;59.4]**
	***p* = 0.002**	*p* = 0.647	***p* = 0.003**	***p* = 0.001**	*p* = 0.615	***p* = 0.001**
	*R*^2^ = 0.509	*R*^2^ = 0.534		*R*^2^ = 0.638	*R*^2^ = 0.663	
CRP (mg/L)	***B* = 3.0**	*B* = 11.1	*B* = *2.6*	***B* = 3.0**	*B* = 11.0	B = 2.6
	**[0.8;5.3]**	[– 13.2;35.4]	*[– 0.1;5.3]*	**[0.6;5.4]**	[− 14.3;36.4]	[− 0.2;5.4]
	***p* = 0.013**	*p* = 0.343	*p* = *0.057*	***p* = 0.017**	*p* = 0.361	*p* = 0.068
	*R*^2^ = 0.368	*R*^2^ = 0.392		*R*^2^ = 0.377	*R*^2^ = 0.401	
Fibrinogen (mg/dL)	***B* = 9.1**	*B* = 40.1	*B* = 7.4	***B* = 9.4**	*B* = 40.5	*B* = 7.7
	**[0.5;17.6]**	[– 51.4;131.6]	[– 2.7;17.5]	**[1.6;17.3]**	[− 42.9;124.0]	[− 1.5;16.9]
	***p* = 0.039**	*p* = 0.361	*p* = 0.137	***p* = 0.022**	*p* = 0.311	*p* = 0.092
	*R*^2^ = 0.270	*R*^2^ = 0.299		*R*^2^ = 0.441	*R*^2^ = 0.471	

Bold: Statistically significant

Italic: not fully significant

Data derived from spline regression analysis

*LDH* lactate dehydrogenase, *CRP* C-reactive protein

**Fig. 2:**
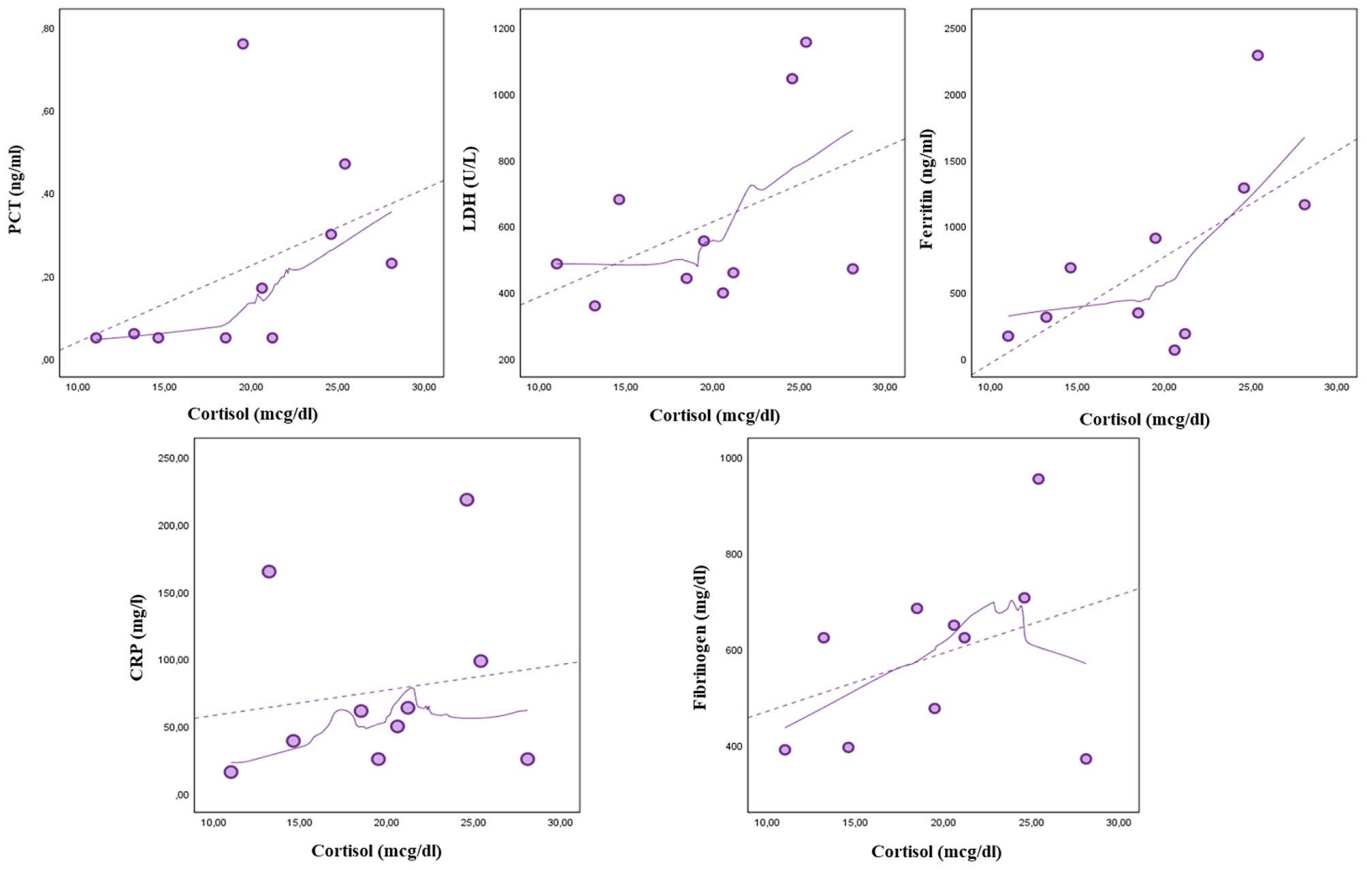
Relationship between cortisol and biochemical inflammatory markers of severity of SARS-CoV-2 pneumonia. The smooth curves were carried out by locally weighted scatterplot smoothing (LOWESS) analysis. *PCT* procalcitonin, *LDH* lactate dehydrogenase, *CRP* C-reactive protein

**Fig. 3:**
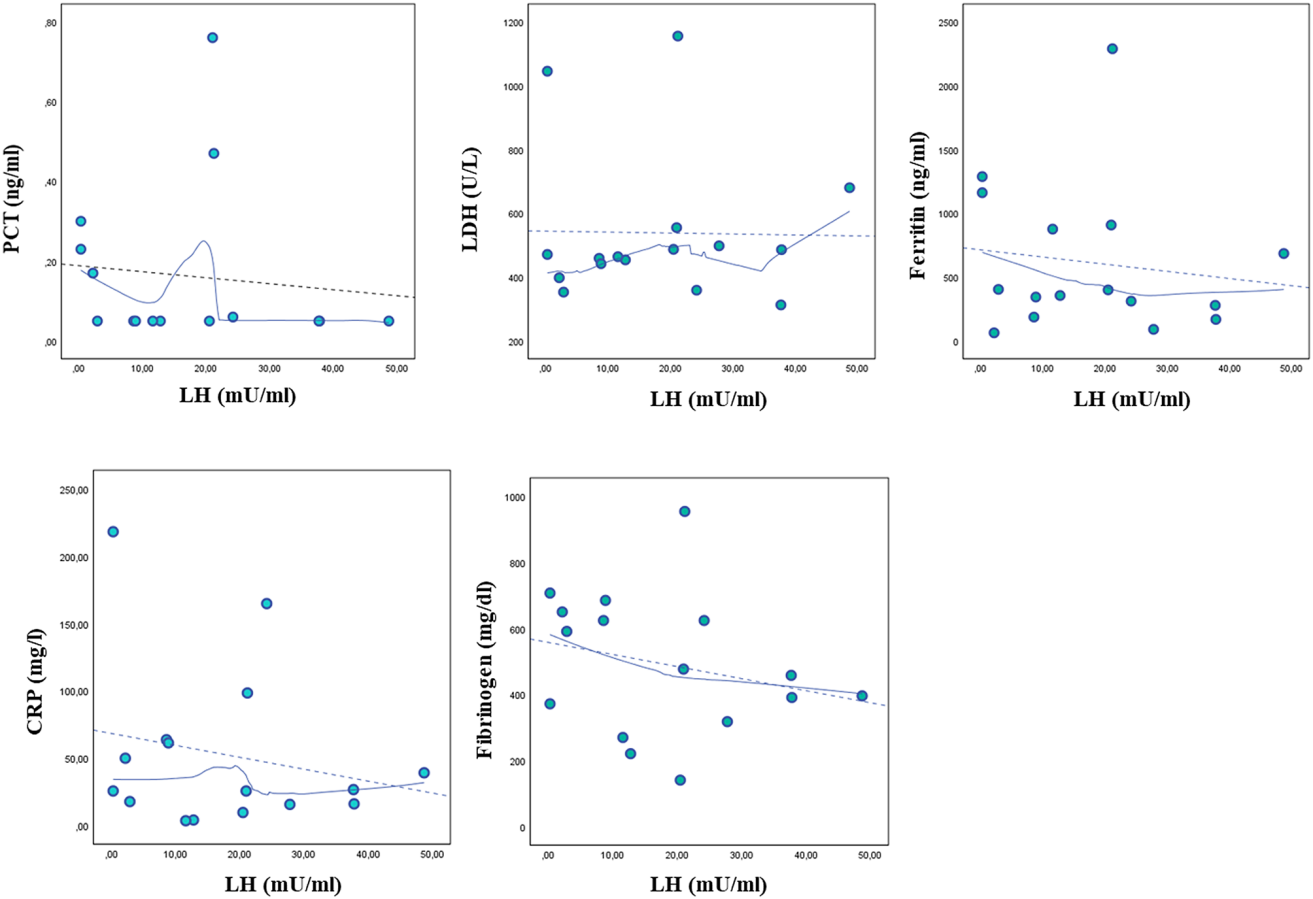
Relationship between LH and biochemical inflammatory markers of severity of SARS-CoV-2 pneumonia. The smooth curves were carried out by locally weighted scatterplot smoothing (LOWESS) analysis. *PCT* procalcitonin, *LDH* lactate dehydrogenase, *CRP* C-reactive protein

LOWESS curves were carried out for the association between cortisol or LH with the same inflammatory parameters (Figs. 2 and 3, respectively). Figure 2 shows that cortisol is associated with serum inflammatory markers with similar trends observed for TT although without reaching statistically significant relationships; conversely, as shown by Fig. 3, the relationship between LH and inflammatory markers had different trends than those observed for TT and cortisol.

When comparing women with different ARDS severity, we confirmed that those with severe ARDS had significantly higher TT than women with moderate ARDS (1.5 [0.4–1.7] vs. 0.4 [0.1–1.3] nmol/L, *p* = 0.05). No differences were found in LH and cortisol levels.

During the follow-up period, only one of the observed women with SARS-CoV-2 pneumonia deceased. She was 83 years old and her hormone values on the first morning after RICU admission were as follow: TT = 1.25 nmol/L, albumin = 3.0 g/dL, SHBG = 14.8 nmol/L, cFT = 40.4 pmol/L, LH = 0.3 U/L, cortisol = 24.6 mcg/dL. She deceased after 6 days in RICU. The remaining patients were transferred to internal medicine clinic for improved clinical conditions after 6.0 [3.3–12.8] days in RICU.

## Discussion

Our study evaluates for the first time the role of testosterone in a cohort of women with SARS-CoV-2 pneumonia. Despite based on a small sample size, the present results show that higher TT and cFT are associated with a greater severity of the disease, as suggested by higher serum pro-inflammatory markers and longer stay in RICU before the transferral to lower intensity care units.

This is in complete opposition to recent findings in men from the same [10] and other, although similar, clinical settings [15–17], which showed that low rather than high T level is associated with worse inflammatory marker profile and poorer clinical outcomes.

The role of T in inflammation in men and women is a challenging and still incompletely unraveled topic for Gender Medicine. In both animal models and men [18, 19], low T is associated with organ-specific as well as with systemic inflammation, as denoted by a rise in pro-inflammatory cytokines [20, 21]. Accordingly, T therapy is able to improve the inflammatory features and to decrease circulating pro-inflammatory markers [22]. The evidence in female is scanty and contradictory. In women with Polycystic Ovary Syndrome (PCOS), the androgen excess is associated with a pro-inflammatory status [23, 24]. Furthermore, in the context of some autoimmune diseases such as rheumatoid arthritis, women with lower number of CAG repeats in the androgen receptor gene, that confers higher androgen sensitivity, develop a more severe clinical course [25]. On the other hand, androgen treatment has been associated with clinical improvement in some autoimmune disorders. In post-menopausal women with active rheumatoid arthritis, 1 year of treatment with 50 mg of T propionate every two weeks resulted in improved general wellbeing [26]. Similarly, 12 weeks of treatment with DHEA 200 mg daily, as compared with placebo, improved the clinical manifestations of systemic lupus erythematosus in women receiving also standard therapy [27]. Recently, the anti-inflammatory mechanisms of T have been studied in vaginal smooth muscle cell from rats and humans. The activation of the androgen receptor by the androgen receptor super-agonist DHT inhibits the secretion of several pro-inflammatory factors while counteracting the chronic and self-perpetuating inflammatory features [28]. Overall, these data indicate how complex- and still not completely unravel—the role of T is in women.

While the results in SARS-CoV-2 infected men described in our [10] and other cohorts [15–17] are in keeping with the expected anti-inflammatory role of T, the results hereby described on SARS-CoV-2 infected women are in apparent contrast. The increased levels of pro-inflammatory markers that are associated with higher T levels suggest a pro-inflammatory effect of T in this population.

In the attempt of hypothesizing possible mechanisms for explaining this observation, we analyzed the hormone levels in deeper detail. A noteworthy result, emerging from the observation of this cohort, is that T levels are surprisingly high for menopausal women. In women, T is mainly secreted by the ovary, driven by LH stimulation of theca cells in the reproductive age. In the post-menopausal period, similar to estradiol or other ovarian sex hormones, T levels physiologically decline [29]. The post-menopausal decline in ovarian hormones is accompanied by an increased gonadotropins level. It is therefore surprising that, in this cohort of women, relatively low LH levels were observed. In fact, one out of four women admitted to RICU had LH levels below 4.3 U/L (lower limit of the interquartile range) largely below the values expected in menopausal women. Therefore, it could be hypothesized that the high level of T in women with COVID-19, being strongly associated with cortisol level, could be mostly produced by the adrenal gland. High testosterone from the adrenal source, in turn, will then exert a negative feedback on pituitary, resulting in an inhibitory effect on the post-menopausal-associated LH increase. However, low LH could reflect not only the T-related negative feedback but also the direct suppressive effect of systemic inflammatory status itself on gonadotropins. Noteworthy, the adrenal source of T in women is often overlooked and considered negligible. However, its role may get important in stressful conditions, such as those related to acute and critical illnesses, including i.e. SARS-CoV-2 pneumonia, which might induce an adrenal cortex hyperactivity and cortisol surge. The hyperactivation of the hypothalamic–pituitary–adrenal axis is not specific to the zona fasciculate because it activates also the zona reticularis with a potential overproduction of androgens. The tight positive association of circulating T with cortisol levels, whilst lacking an association with serum LH, further substantiate the hypothesis of an adrenal origin of the increased T level. Moreover, stress-related cortisol excess may also explain the inappropriately low LH levels; a significant negative relationship links cortisol and LH in the present cohort. If confirmed, the observed association between T and cortisol level may be of paramount importance since cortisol has been recently claimed as a better independent predictor of an increased mortality in COVID-19 patients than other laboratory markers [30]. In this large cohort of COVID-19 patients admitted to three teaching hospitals in London UK (*n* = 535), baseline cortisol concentration higher than 744 nmol/L was predictive of a reduced median survival [30]. In our study cohort, which is characterized by a greatly lower sample size, we were not able to select an optimal cut-off for cortisol, as opposed to TT and calculated free T.

The data hereby presented suggest that, in a severe acute and critical illness, such as SARS-CoV-2 pneumonia, the inflammatory response elicits the activation of stress hormone response, as suggested by increased cortisol levels. The relatively high T levels in menopausal women are, similar to cortisol, the expression of the adrenal cortex activation. This explains the relationship between higher T levels and increased inflammatory markers. Therefore, it might be conceivable that T is a mirror, more than a pathogenic factor, of the inflammatory burst in SARS-CoV-2 women. Nevertheless, as the cross-sectional design of the study does not allow defining the cause-effect relationship, a contribution of T in worsening COVID-19 related inflammatory status could not be ruled out. The transmembrane serine protease 2 (TMPRSS2) that is involved in the proteolytic processing of the SARS-CoV-2 spike protein thus facilitating the viral entry within the cells, has been previously studied in malignant prostatic tissue [31]. In this context, TMPRSS2 has been proven as an androgen dependent protein [31] with androgen receptor as a promoter for TMPRSS2 gene transcription [32]. Accordingly, it may be speculated that higher testosterone levels in this study population may contribute to cause a more severe disease also through this pathogenic mechanism.

An understanding of the gender-related differences in adrenal hyperactivation during SARS-CoV-2 infection would be noteworthy. In men, increased T of adrenal source is not documentable because testis activity, although impaired in SARS-CoV-2 infection [10, 15–17], dilutes the effect and, eventually T appears as very low for adult males.

Some limitations should be recognized. Firstly, the sample size is very small and the results here reported need to be interpreted cautiously and confirmed in wider populations. For the moment, only one non-peer-reviewed report has been published on a cohort of SARS-CoV-2 women with a similar sample size and characteristics, which found results comparable with ours [33]. Further studies are advocated. Moreover, for simplicity’s sake, only a single baseline testosterone and cortisol concentration measurement was analyzed, thus without accounting for intra- and interindividual variations of hormones response to stress. Thirdly, among our women, only a patient experienced an adverse outcome. This prevents from evaluating the predicting role of T on clinical outcomes; however, her hormone levels (high cortisol, inappropriately high T and low LH for an 83-years old woman) are in keeping with our hypothesis. Moreover, the study is a retrospective one and information on ACTH, or other adrenal glands hormones i.e. androstenedione, DHEAS and 17OH-progesterone are not available. These would have been useful to support or disprove our hypothesis. However, we found a tight association between cortisol level and all the pro-inflammatory markers, but a threshold for predicting a worst clinical presentation or course was not found. In addition, T was not assessed by the gold standard method. However, the commercially available immunoassay used in a high-volume hospital, which undergoes quality control programs makes the measurement reliable. Finally, information on the onset of infection before RICU is not available.

In conclusion, opposite to men, in women with SARS-CoV-2 pneumonia, higher TT and cFT are associated with a stronger inflammatory status. In line with the menopausal status of the enrolled women, T seems not to be of ovarian origin. Adrenal cortex hyperactivity, secondary to a stressful critical ill condition, may be hypothesized as T source. Further larger and specifically designed studies are necessary to confirm these preliminary data.

## Data Availability

The datasets generated during and/or analyzed during the current study are available from the corresponding author on reasonable request.
